# Food Safety Challenges towards Safe, Healthy, and Nutritious Street Foods in Bangladesh

**DOI:** 10.1155/2014/483519

**Published:** 2014-04-08

**Authors:** Md. Khairuzzaman, Fatema Moni Chowdhury, Sharmin Zaman, Arafat Al Mamun, Md. Latiful Bari

**Affiliations:** ^1^Center for Advanced Research in Sciences, University of Dhaka, Dhaka 1000, Bangladesh; ^2^Department of Microbiology and Biotechnology, Jagannath University, Dhaka 1000, Bangladesh

## Abstract

The street foods play an important socioeconomic role in meeting food and nutritional requirements of city consumers at affordable prices to the lower and middle income people. The number of food poisoning notifications rose steadily worldwide since the inception of *E. coli* O157:H7 outbreak in the 1980s to date. This may be partly attributed to improved surveillance, increased global trade and travel, changes in modern food production, the impact of modern lifestyles, changes in food consumption, and the emergence of new pathogens. Consumer's knowledge and attitude may influence food safety behavior and practice. For the sake of public health, it is important to understand the epidemiology of foodborne illnesses that help in prevention and control efforts, appropriately allocating resources to control foodborne illness, monitoring and evaluation of food safety measures, development of new food safety standards, and assessment of the cost-effectiveness of interventions. This review paper described the sociodemographic characteristics, common hazards, and occupational hazards of street food vendors, microbial risk associated with street food, food safety interventions and control measures, regulatory aspects and legal requirements, financial constraints, and attitudes.

## 1. Introduction

The street foods play an important socioeconomic role in meeting food and nutritional requirements of city consumers at affordable prices to the lower and middle income groups and are appreciated for their unique flavors and convenience [1–3]. Street foods also assure food security for low income urban population and livelihood for a significant proportion of the population in many developing countries. Street foods are described as wide range of ready-to-eat foods and beverages or prepared at home and consumed on the streets without further preparation [4]. These food items are usually sold by vendors and hawkers in the streets or other similar public places. While street vended foods are appreciated for their unique flavors as well as their convenience, they are also important in contributing to the nutritional status of the population. In contrast to these potential benefits, it is also recognized that street food vendors are often poor, uneducated, and lack knowledge in safe food handling, environment, sanitation and hygiene, mode of food display, food service and hand washing, sources of raw materials, and use of potable water. Consequently, street foods are perceived to be a major public health risk [5].

Foodborne illnesses of microbial origin are a major health problem associated with street foods [6–8]. In addition, resistance of foodborne microorganisms in multi-drug made the food safety situation more vulnerable in public health [9]. Approximately, 30 million people in Bangladesh are suffering from foodborne illnesses each year [10]. Diarrheal diseases are the most common food poisoning cases in Bangladesh and in some cases, these can cause death. The diseases are caused by either toxin from the microbe or by the human body's reactions to the microbe. The traditional processing methods that are used in the preparation, inappropriate holding temperature, and poor personal hygiene of food handlers are some of the main causes of contamination of street foods [11, 12]. Also the foods are not effectively protected from flies and dust [13, 14]. In Bangladesh, street foods are mostly prepared and processed manually and sold to the public at various lorry terminals, by the roadside or by itinerant vendors [8]. A study of the socioeconomic conditions and determination of the hygienic and sanitary practices of street food vendors in Dhaka City Corporation was carried out by FAO 2010 [15]. The study result demonstrated that 25% street food vendors are illiterate and cannot write their names and have no formal education. As street food business requires low investment, most of the vendors (88%) were found to own the business. They reportedly work for 13–18 hours a day without having toilet facilities. Most of the vending shops (68%) were located on the footpath irrespective of areas surveyed and 30% vending carts were placed near the municipal drain and 18% near the sewerage. Microbiological study of different foods items, drinking water, and hand swab samples showed the prevalence of overwhelmingly high numbers of aerobic bacteria, coliform bacteria, and pathogens [15].

For the sake of public health, it is important to understand the epidemiology of foodborne illnesses because it will help in prevention and control efforts, appropriately allocating resources to control foodborne illness, monitoring, and evaluation of food safety measures, development of new food safety standards, and assessment of the cost-effectiveness of interventions. The purpose of this study is to see the microbial risk of food poisoning associated with street food in order to determine the magnitude of the problem, risk factors, monitoring and surveillance, and measures of control.

## 2. Sociodemographic Characteristics of Street Food Vendors

The street food vendors of Bangladesh are not enumerated in the formal sector of country's economy. They are identified as the informal sector where their businesses are conducted as a form of irregular, unstable, and marginal economic activities. As such there is no systematic documentation of the numbers of street food vendors, their scale of businesses, or the viability of their pursuits. After rickshaw-pulling, street vending is probably the second most important employment opportunity for the urban poor in Bangladesh, and particularly important for young and middle-aged men who have migrated to Dhaka in the past five to ten years [16]. Roughly  750,000 rickshaw pullers and 300,000 street vendors live and work in Dhaka [17]. Dhaka is among the world's cities with the highest number of hawkers: in Asia, only Mumbai (~250,000), Delhi (~200,000), Calcutta (~150,000), and Bangkok (~100,000) have similarly large numbers of street vendors [5]. It is impossible to establish the absolute number of street food vendors in Dhaka. However, Benjamin [18] conducted a survey on street food vendors in Dhaka, over a period of three years (2007 to 2010). This survey and official labour statistics demonstrated that between 90,000 and 100,000 street vendors sell prepared food items, and around 418,000 people or 2.9 percent of Dhaka's total population depend on the income generated by street food vendors. This data indicates that each vendor serves 84 customers per day on average. This implies that almost eight million people or 55 percent of the population of Dhaka take some street food everyday [19]. The significance of street food system of Dhaka is beyond doubt and selling street food is not a marginal economic activity, but a normal—yet highly visible—social practice, that is, economically efficient and deeply embedded in the urban economy and in urban life [2, 4, 20]. A glimpse of the socioeconomic background of the vendors is presented below to help understand who the street food vendors are.

- Both males and females and married and unmarried operate as street food vendors. Their age range is between 25 and 60 years with a majority being in the age group of 30–40 years.
- Many street food vendors and their families have their origin in rural backgrounds or have moved to urban centers at a later stage or else live in rural areas and travel daily to the city for their business operations.
- The level of education achieved by the street food vendors is comparatively low and in the case of a majority, education levels varied between grades 5 and 8.
- Many street food vendors are constrained by the unstable socioeconomic backgrounds in their families.
- Employment history of the street food vendors shows their previous involvement in several urban-based, irregular, and low-paid income generating activities, which required hard manual labor, prior to their involvement in the street food business. Their engagement in such activities was not sufficient for their sustenance. The result was that the vendors moved from one work place to another.

Street food vendors are a self-employed category of small entrepreneurs who are not dependent on any institutional structures to find their livelihoods. Their enterprises evolve exclusively around their own individual strengths and the support extended to them by their immediate social networks such as family members and other close associates. The earnings from their business enterprises are a means of living for the vendors themselves and their dependent family members. As such, these economic activities of the street food vendors have not only provided a source of livelihood to the vendors and their dependent family members but also have reduced the plight of their becoming an economic and social burden on the state.

Street food satisfies the food consumption need of a significant section of the population. The food sold on the streets is relatively cheap and readily available. It is sometimes brought to the door step of the customers. Street food, therefore, not only meets the food requirements particularly of those of the low income categories but also the busy customers who do not have much time either to prepare their own food or to go to other eating houses where probably the food is more expensive and servicing is time consuming.

## 3. Working Conditions and Occupational Hazards of Street Food Vendors

Street vendors face unique kinds of livelihood risks because of the legal, physical, and sociocultural environment in which they work. The most pressing and ongoing risk for many street vendors is the possibility that local government authorities will forcibly remove them from the streets or confiscate their merchandise. This risk of displacement often increases in the context of elections, mega events, or efforts to beautify historic city centers. Just like formal business operators, street vendors are less productive in unstable institutional environments where rules are irregular and unpredictable [5, 19].

Street traders face more routine occupational hazards as well. Many must lift and haul heavy loads of goods to and from their point of sale each day. The physical environments in which they work typically lack proper infrastructure, such as clean running water, toilets, and solid waste removal (Table 1). Street vendors are exposed to physical harm from the improper provision of fire safety equipment and the improper regulation of traffic in commercial areas. They are also exposed to a high concentration of air pollutants and to inclement weather. These physical risks take a particular toll on young children who must accompany their mothers to vend in the streets. Given the growing numbers of the street food vendors and the customers who patronize them, the issues and problems the vendors encounter need special attention of the authorities concerned. Income and earning risks are also common to many street vendors. Harassment on the part of local authorities—including evictions, confiscation of merchandise, and demands for bribes—is a common source of income risk for street vendors [21].

**Figure 1:**
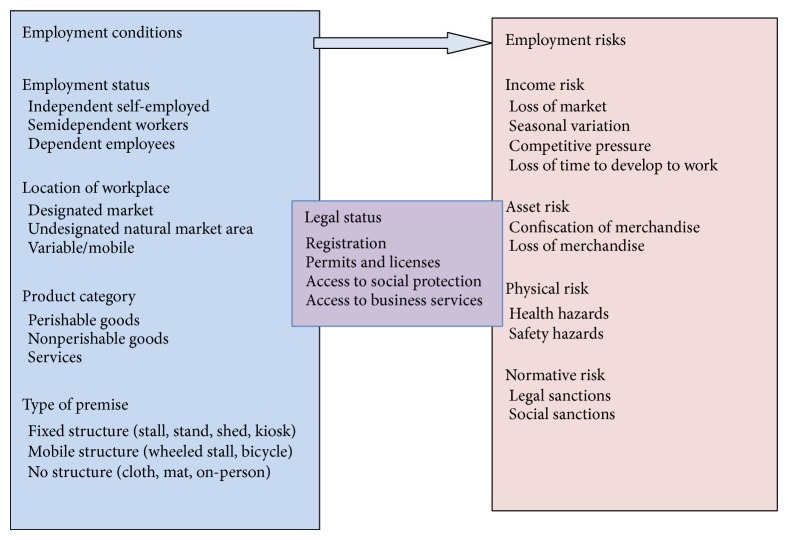
Employment condition and employment risk among street vendors, the number of vendor surveyed was 1137 (adapted from http://wiego.org/informal-economy/occupational-groups/street-vendors) [19].

Street vendors legal status can act as a bridge between their employment conditions and the range of employment risks they face (Table 1 and Figure 1). A vendor with a fixed structure in a designated market, for example, may be more likely to hold a license or permit, and in turn would be less exposed to certain kinds of risks. Likewise, a street vendor who works as an employee selling a particular kind of product, such as newspapers, may be better protected by law and therefore less vulnerable. Obtaining legal status of some kind is therefore a key demand of street trading organizations in many cities.

## 4. Growing Demand of Street Food

The urban population in Bangladesh is increasing rapidly. In the last decade, the number of people living in the country's capital Dhaka almost doubled from 5.3 to 9.3 million [22]. This development has led to an increase in the demand for relatively inexpensive and ready-to-eat foods as many urban residents spend most of the day outside of the house and have little time and money to spend on food. Rapid urbanization also turned street-food vending into an important business; in Dhaka alone, around 200,000 people earn their living by selling street foods [4, 23]. The low cost, accessibility, and convenience are the key factors for the growing popularity of street foods. Women play a very vital role in the street food sector through their direct and/or indirect involvement in the business. Additionally a significant number of street vendors are woman-headed households [1, 19]. The diversity that exists among street food vendors is reflected in the type of food they prepare/sell, the scale of their business, the mode in which they are operating, the locations in which they prepare and sell food, the type of clients to whom they sell food, and so forth. Street food ingredients are area-specific and mostly undocumented. There are so many varieties that it is impossible to provide a menu of all the different street foods consumed around the world. In Bangladesh, street foods include chola boot (chickpeas), bhelpuri (puffed rice with potatoes), and samucha (deep-fried dough stuffed with vegetables and/or meat) as well as drinks like sugar-cane juice and lassi (yoghurt and water). Other popular snacks are ghugni (boiled and mashed white peas with spices), singara (flour wraps stuffed with vegetables, spices, and occasionally liver), and different types of cakes [7].

## 5. Consumers of Street Food

The consumption patterns of street foods and their contribution to dietary intake are scanty. The customer surveys undertaken by FAO 2006 [24] and other investigators revealed that the main consumers of street foods in most countries were other members of the informal sector, such as fellow hawkers and hustlers and casual wage laborers. Other important categories of customer were children and students, office workers, and housewives [25]. The studies also found that street foods were consumed across all income groups and the proportion of the daily household food budget spent on street foods was high, ranging from 25 percent in Bogor to 47 percent in Chonburi, Thailand [23]. The frequency and regularity of consumption were variable: in some countries, street foods were bought daily and formed an integral part of the diet; in others, notably in Bangladesh, they appeared supplementary and few customers bought them daily [6, 26]. Some categories of consumer (students, itinerant unskilled laborers, and the homeless) were found to buy almost all their food from vendors [23]. The cost of street foods is usually competitive compared with that of foods purchased from larger food establishments, such as restaurants and fast food outlets. Also, due to the sometimes high costs of fuel and ingredients in urban contexts, economies of scale can create a street food cheaper than the same food prepared at home.

## 6. Safety of Street Foods

The hygienic aspects of street food vending are a major concern for food control officers. Vending stands are often crude structures, and running water, washing facilities, and toilettes may not be available. Improved safety of street foods can be achieved through awareness raising programmes involving several partners such as local authorities, the food vendors, government departments, consumer organizations, standard setting bodies, and some nongovernmental organizations. In some instances, the vendors are keen to participate in programmes that provide basic facilities that make it possible for them to work in clean environments. For example, in a survey of street food vendors in Lusaka and Harare, the vendors indicated that they would be willing to pay for basic facilities such as running water and electricity but would want the local authorities to provide the water points, refuse receptacles, and washing facilities [27]. A viable partnership involving local authorities, vendors and policy makers is therefore encouraged as this should lead to the improvement of business conditions and allow for the improvement of the livelihoods of vendors and their families.

### 6.1. Microbiological Safety

The battle against foodborne diseases is facing new challenges due to the globalization of the food market, climate change, and changing patterns of human consumption as fresh and convenient foods are currently preferred [28]. As food is biological in nature, it is capable of supporting the growth of microorganisms and foodborne diseases result from the ingestion of contaminated foods and food products [29]. More than 250 different types of viruses, bacteria, parasites, toxins, metals, and prions are associated with foodborne diseases in humans [30]. Although viruses are more responsible for more than 50% of all foodborne illnesses; generally hospitalizations and deaths associated with foodborne infections are due to bacterial agents. The infections range from mild gastroenteritis to life-threatening neurologic, hepatic, and renal syndromes caused by either toxin from the disease-causing microbe or by the human body's reaction to the microbe itself [31].

Foodborne bacterial agents are the leading cause of severe and fatal foodborne illnesses. Of the many thousands different bacterial species, more than 90% of food-poisoning illnesses are caused by species of* Staphylococcus, Salmonella, Clostridium, Campylobacter, Listeria, Vibrio, Bacillus, *and Enteropathogenic* Escherichia coli *[32]. For instance, in the US and France, in the last decade of the 20th century,* Salmonella *was the most frequent cause of bacterial foodborne illness (5,700–10,200 cases), followed by* Campylobacter *(2,600–3,500 cases) and* Listeria *(304 cases) [33]. In South Africa, species of* Listeria*,* Enterobacter, and Aeromonas *were the most prevalent bacteria in ready-to-eat foods [34].

However, no such databases are available for Bangladesh as well as other developing countries. Therefore, from October 2012 to the present, our laboratory conducted a series of experiments to assess the microbial quality of street foods of Bangladesh [7]. More than 100 street foods samples of 20 kinds including singara, jhal-muri, chatpati, chetoi pitha, chola/bengal gram, jilapi, jar drinking water, pickles, amra, tehari, vegetable rolls, sugarcane juice, raw cucumber slices, milk, other juices, beverages, and bread were analyzed for major foodborne pathogens including,* Salmonella* spp.,* Escherichia coli* O157, O111, O26, and other* E. coli*, other coliforms,* Listeria* spp., and* Staphylococcus* spp. The presence of all the above mentioned pathogenic organisms found in the street foods was presented in Table 2. In addition, the isolated pathogenic organisms were found resistant to at least 7 antibiotics. These studies demonstrated that foods sold in the street of Dhaka city constitute a potential microbial hazard to human health [7]. In addition, Mamun et al. [8] reported that among different items of street vended foods, 54% of sliced fruits samples, 59% of jhalmuri samples, 29% of chotpotis samples, 53% of vajavuji samples, and all (100%) sharbat samples were found unsatisfactory microbial quality. This finding also reflected poor microbiological quality of the school-based street vended foods indicating a health threat to the school children of Dhaka city [1, 8].

### 6.2. Chemical Safety

Nonfood grade chemical additives, such as colorants and preservatives, and contaminants, such as pesticide residues, have also been found in street foods. A chemical analysis of street foods in Bogor found unpermitted coloring agents such as textile dyes and also pesticide residues [4]. Proper use of salt, spices, nitrates, and sugar is an important means of preventing food spoilage, but the drive to keep prices low may lead to the purchase of cheap ingredients containing unpermitted chemical additives from unauthorized suppliers. Chemicals such as colorants may also be added to mask the poor quality of cheap materials.

Due to the conditions under which street foods are sold, there is concern that food may be contaminated with heavy metals and pesticide residues. These contaminants may come from the utensils, raw materials, or transport methods used and may also occur due to the lack of appropriate storage facilities [29].

A study carried out in Ghana revealed that street food vendors source their pots and other utensils from both formal and informal manufacturers/retailers. Some of the street food samples had higher levels of  lead, cadmium, arsenic, mercury, and copper than average food samples, suggesting possible leaching from the utensils [35]. Further tests showed that lead from the pots obtained from informal manufacturers could leach into the food. These pots are manufactured using scrap metal that could come from diverse sources such as derelict cars, car batteries, and industrial machinery, which are obviously not suitable for use with foods. Their continued use must be discouraged. Interviews with vendors also showed that some of their utensils come from informal sources [11]. This was attributed to the fact that when police raid these vendors, they usually confiscate their wares, including the pots and utensils. For fear of  losing their more expensive pots, the vendors resort to using informally fabricated pots, thereby exposing consumers to the possibility of food contamination by heavy metals. Further work must be done in order to reduce the exposure of consumers to heavy metals and pesticide residues through street-vended food [29, 36].

### 6.3. Personal Hygiene

Purchasing ready-to-eat foods and ingredients from street/market vendors poses a considerable risk to public health, especially due to the poor hygienic practices. In most cases, the vendors do not have adequate washing facilities, and some vendors started their duties without taking a proper bath. Some of the vendors sleep at the vending sites in order to protect their wares [27]. Foods and ingredients are also subjected to repeated contamination from unwashed hands and the materials used for wrapping, such as leaves, old newspapers, and reusable polyethylene bags [37].

However, many vendors are aware of the need to wear clean and appropriate clothes. Some of the female vendors wear headgear and aprons. After a few awareness-raising campaigns for vendors, most of them understand the need to have clean clothes and utensils [11]. But the absence of water points near their work places and poor drainage facilities make them unable to practice good hygiene. Moreover, some food handlers washed their hands in the same bucket used for cleaning utensils, which may lead to the contamination of food with faecal matter. On the other hand, most food vendors operate their business without health certificates or licenses, which poses additional concern and required extensive training programme on food preparation and handling techniques [22].

Street food vendors use cheap bar soap than liquid soap, which may be more effective, to clean their utensils, but as they use cold water, resulting in inefficient cleaning. Furthermore, washed plates are often stored in an unclean corner, plastic bowl, or cardboard box, leading to recontamination of the plates.

### 6.4. Environmental Hygiene

Inadequate refuse disposal facilities lead to the accumulation of refuse at food vending sites. This leads to an increased pest population and resulted in an increased risk of food contamination. In many instances, the vending sites are not included within the city or town plans, and therefore amenities such as refuse collection are not available. City authorities are often faced with the dilemma that if they provide services to illegal operations, this will imply recognition of these operations. At the same time, because the vending operations are illegal and vendors do not contribute anything towards the maintenance of infrastructure or provision of public services, therefore, they are not entitled to the service. This contributes to further deterioration of the hygienic condition of the area where the foods are vended [38]. Poor sanitary conditions in the area where foods are vended also contribute to poor food storage and transport conditions. Street food vendors in some cities obtain their vegetables, maize meal, and other condiments from licensed shops, and therefore there is less concern regarding the safety of these raw materials. However, most of the vendors have no fixed stalls where they can store their raw materials on site. They usually store their goods at home overnight and transport them the following day, often improperly covered, to their operating sites. Thus, the food becomes prone to contamination during transportation [18, 19].

## 7. Food Legislation and Regulatory Aspects

Food legislation and regulatory control of street foods varies from country to country. A recent review of the situation in Asia found great diversity among the legal instruments developed to control the street food trade. Some countries had no specific legislation or control systems at all [19, 22]. In those countries where street food activities were regulated by law, the regulations or by-laws affecting the street food trade were part of a larger body of legislation dealing with food, health, or environmental sanitation. Licensing or registration systems, inspection systems, and codes of practice are other forms of regulation that are in effect in some countries.

A number of pieces of legislation relating to the preparation and sale of safe street foods have been established by the Bangladesh government. The Bangladesh Pure Food Ordinance 1959 (revised 2005) has several sections dealing with the safety of street food: adulteration of food; prohibition of calcium carbide, formalin, and insecticide; selling unwholesome food; uncovered foods; and unhygienic premises and violations of the health code. The other relevant legal measures related to safe street foods are the Bangladesh Standards and Testing Institution (BSTI) Ordinance 37 of 1985; the Consumers Rights Preservation act 2009; and the Penal Code of 1860, sections 272–276.

A mobile court to monitor street food vendors was introduced in the city of Dhaka in 2011 and popularized through the media. A number of civil society organizations have emerged in recent years in Bangladesh to promote safe street foods and overall food safety. For example, the Consumers Association of Bangladesh (CAB), VOCTA (consumer), which has conducted street food surveys and organized awareness, campaigns through rallies, seminars, workshops, and policy advocacy. The electronic and print media are also involved in providing public awareness of safe street foods [15].

In contrast, key constraints to the effective management of street foods are the lack of awareness of personal hygiene and safe food among street food vendors and consumers; insufficient awareness among the consumers about the Consumer Rights Act; lack of clarity in existing legislation and standards on street food; no specific regulatory body for licensing, provision of ID card, medical fitness, or dress codes concerning street food vendors; no demarcation of specific areas by local municipalities for street food vendors; insufficient number of sanitary inspectors; the inability of sanitary inspectors to take penal actions against street food vendors; and absence of appropriate training and supervision of street food vendors [16].

## 8. Conclusion

The quality and safety of street foods is determined by numerous factors such as the business organization, regulatory aspects, technical aspects related to the preparation, preservation and display of food sold in the streets, the consumer perspective, and educational programs. In order to improve the conditions of street food vendors and to make sure that the food sold does not jeopardize public health, the first and foremost necessity is to build awareness that food vendor should maintain certain quality standard. In many areas, street foods are sold and food safety issues are not taken into consideration neither on the producer nor on the consumer side. Consumers tend to look mostly at the price and might be already accustomed to the taste of unhealthy meals. Vendors, on the other hand, have a very small margin of profit and are incentivized to keep expenses low by utilizing low quality ingredients and disregarding costly hygienic practices.

To break this vicious cycle, governments need to embrace street food vendors as a dynamic economic sector. With their adaptability to the frenetic life in the global cities, street food vendors have a huge potential to quickly fill niches, greatly improving urban access to food. While excessive regulation of the sector carries the risk of suffocating this adaptability and would just shift the problem to a new informal sector consisting of those dodging the regulation, certain minimum standards, especially related to food quality, need to be enforced. Vendors should be given some basic training on how to safely prepare and store food and businesses should be certified accordingly.

While some proposed the application of HACCP standards, others argued against it stressing the need for much simpler guidelines such as the five keys to safer food. In addition, municipalities should provide vendors with appropriate infrastructure like access to clean water and sewage systems. Street food vendors should be encouraged to partake in awareness raising programmes and given access to microcredit. In order to improve the vendors standing, strengthening their overall position vis-à-vis authorities, promoting their organization into cooperatives has been identified as a path to follow. In addition to helping vendors run their business in a more efficient and safe manner, cooperatives would also ease the authorities work in enforcing hygienic and business standards.

In general, interventions and programmes can only be successful if they do not focus on one aspect alone. Tackling only food quality, for instance, cannot ensure that street food vendors play the most positive role in realizing food security of the urban population. It is important not to forget that the street foods constitute a very heterogeneous sector and the interventions need to be carefully planned by keeping different aspects such as gender, secondary audience, and local customs into consideration. It is also necessary to differentiate between vendors selling freshly prepared food on the spot or hawking dishes prepared earlier at home, with the second practice being much more risky in terms of foodborne pathogen and spores. Needless to say, general education levels also play an important role in ensuring safe street foods. The more both vendors and patrons will be educated and the more they will know about issues such as nutrition and food safety, the more they will be interested in having the business as clean and the products as healthy as possible.

## Figures and Tables

**Table 1:** Summary of street-vended food in Dhaka city survey, conducted during April to October 2010 among 1137 vendors.

	% answered	Number of vendor
Socioeconomics of street food sector		
Are street-vended foods a significant part of the urban food supply?	74	841
Is street-vending of foods an important mean of employment?	69	784
Are a significant number of women employed in this sector?	62	704
Are street-vended foods important in rural areas?	22	250
Description of street vended foods	% answered	
Type of food		
Grain and cereal	64	727
Fruit and vegetables	86	977
Meat and fish	85	966
Fried foods	76	864
Beverages	65	739
Type of preparation		
Ready to eat	77	875
Cooked on site	82	932
None (raw)	65	739
Condition of preparation		
Preparation too far in advance	73	830
Left at room temperature	82	932
Foods cooked in large pots	33	375
Improper warm holding	72	818
Extra large quantities prepared	38	432
Inadequate reheating	72	818
Inadequate cleaning of equipment	72	818
Conditions normally encountered in the trade	% answered	
Type of facility		
Mobile carts	75	852
Fixed stalls	88	1000
Improved food centers	32	363
Type of infrastructure		
Potable water	47	534
Toilets	15	170
Hand washing facilities	22	250
Dish/utensil washing	48	545
Refrigeration available	None	None
Waste disposal	47	534

	Answered no	Answered yes

Regulation of street foods		
Laws and regulations covering street-vended food exist	1080	56
Registration of street vendors required	113	1023
Code of practice applied	1080	57
Periodic training required	57	1080
Periodical medical exam required	2	1135

**Table 2:** Total aerobic bacterial and total coliform population and presence of different pathogens in the street food samples of Dhaka city.

Street food samples	Total aerobic bacterial population (log⁡CFU/g)	Total coliform population (log⁡CFU/g)	Presence of *E. coli *	Presence of *E. coli* O157, 0111, 026	Presence of *Salmonella *	*Staphylococcus *spp.	*Listeria *monocytogenes
Singara	8.8 ± 0.02	7.5 ± 0.02	ND	ND	ND	9/9	ND
Muri	7.5 ± 0.05	5.0 ± 0.08	ND	ND	3/9	9/9	ND
Chatpati	8.0 ± 0.06	5.8 ± 0.05	ND	ND	6/9	9/9	ND
Chitoi pitha	7.2 ± 0.05	4.0 ± 0.01	3/9	ND	3/9	9/9	ND
Sola	7.5 ± 0.04	4.8 ± 0.09	3/9	2/9	2/9	2/9	9/9
Jilapi	4.9 ± 0.03	3.3 ± 0.06	3/9	ND	3/9	3/9	ND
Jar water	3.0 ± 0.04	2.5 ± 0.08	8/18	2/18	2/18	6/18	ND
Achar	6.5 ± 0.03	2.0 ± 0.01	ND	ND	ND	3/9	ND
Amra	6.3 ± 0.04	2.7 ± 0.04	3/9	ND	ND	3/9	ND
Tehari	6.8 ± 0.05	2.0 ± 0.01	4/9	3/9	ND	2/9	ND
Vegetable Roll	5.7 ± 0.06	2.6 ± 0.05	2/6	1/6	ND	5/9	ND
Sugarcane juice	6.0 ± 0.04	5.1 ± 0.05	ND	ND	2/3	1/3	ND
Slice Cucumber	6.2 ± 0.01	2.7 ± 0.01	6/9	4/9	3/9	6/9	ND
Homemade Bread	3.9 ± 0.08	<1.0	ND	ND	ND	1/3	ND
Milk	7.2 ± 0.05	4.1 ± 0.03	3/3	1/3	ND	3/3	ND
Juice	3.5 ± 0.03	2.4 ± 0.06	3/3	ND	ND	2/3	ND
Bottled drinks	3.7 ± 0.07	<1.0	ND	ND	ND	ND	ND

Results are expressed in average of three replicate samples ±SD, which are being calculated from duplicate plates. ND: not detected; <1.0: below detection limit; “*x*/*y*” for *x* positive in *y* analyzed samples.

## References

[1] Ackah M., Gyamfi E. T., Anim A. K., Osei J., Hasnsen J. K., Agyemang O. (2011). Socioeconomic profile, knowledge of hygiene and food safety practices among street-food vendors in some parts of Accra-Ghana. *Internet Journal of Food Safety*.

[2] Cross J. C., Morales A. (2007). *Street Entrepreneurs: People, Place and Politics in Local and Global Perspective*.

[3] Muzaffar A. T., Huq I., Mallik B. A. (2009). Entrepreneurs of the streets: an analytical work on the street food vendors of Dhaka city. *International Journal of Business and Management*.

[4] Rane S. (2011). Street vended food in developing world: hazard analyses. *Indian Journal of Microbiology*.

[5] Bhowmik S. (2010). *Street Vendors in the Global Urban Economy*.

[6] Biswas S., Parvez M. A. K., Shafiquzzaman M., Nahar S., Rahman M. N. (2010). Isolation and characterization of *Escherichia coli* in ready-to-eat foods vended in Islamic University, Kushtia. *Journal of Bio-Science*.

[7] Tabashsum Z., Khalil I., Nazim Uddin M., Moniruzzaman Mollah A. K. M., Inatsu Y., Latiful Bari Md. (2013). Prevalence of Foodborne Pathogens and Spoilage Microorganisms and their Drug resistant status in different street foods of Dhaka city. *Agriculture Food and Analytical Bacteriology*.

[8] Mamun M. A., Rahman M. M., Turin T. C. (2013). Microbiological quality of selected street food items vended by school-based street food vendors in Dhaka, Bangladesh. *International Journal of Food Microbiology*.

[9] Ali M., Khan M., Saha M. L. (2011). Antibiotic resistant patterns of bacterial isolates from ready-to-eat (RTE) street vended fresh vegetables and fruits in Dhaka City. *Bangladesh Journal of Scientific Research*.

[10] Food and Agriculture Organization The State of Food Insecurity in the World 2012. http://www.fao.org/docrep/016/i3027e/i3027e00.htm.

[11] Barro N., Bello A. R., Savadogo A., Ouattara C. A. T., Ilboudo A. J., Traore A. S. (2006). Hygienic status assessment of dish washing waters, utensils, hands and pieces of money from street food processing sites in Ouagadougou (Burkina Faso). *African Journal of Biotechnology*.

[12] Mensah P., Yeboah-Manu D., Owusu-Darko K., Ablordey A. (2002). Street foods in Accra, Ghana: how safe are they?. *Bulletin of the World Health Organization*.

[13] Dardano C. Carribbean regional working group on street food vendors. ftp:ftp.fao.org/es/esn/food/carribean_report.pdf.

[14] Muyanja C., Nayiga L., Brenda N., Nasinyama G. (2011). Practices, knowledge and risk factors of street food vendors in Uganda. *Food Control*.

[15] Food and Agricultural Organization of the United Nations Improving food safety, quality and food control in Bangladesh.

[16] World Bank (2007). *Improving Living Conditions for the Urban Poor*.

[17] Islam N. (2005). *Dhaka Now. Contemporary Urban Development*.

[18] Benjamin E. Street Food Governance in Dhaka (Bangladesh): the appropriation of street vending spaces and the informal politics of exploitation.

[19] http://wiego.org/informal-economy/occupational-groups/street-vendors.

[20] Chen M. A. Rethinking the Informal Economy. Linkages with the Formal Economy and the Formal Regulatory Environment. http://www.wider.unu.edu/publications/working-papers/research-papers/2005/en_GB/rp2005-10/_files/78091752824112453/default/rp2005-10.pdf.

[21] Brown A., Lyons M., Dankoco I. (2010). Street traders and the emerging spaces for urban voice and citizenship in African cities. *Urban Studies*.

[22] Consumer Association of Bangladesh (CAB) Institutionalization of Healthy Street Food System in Bangladesh: Safer street Foods: A Pilot Study with Three Wards of Dhaka City Corporation as a Model: 1-2. http://www.nfpcsp.org.

[23] Zain M. M., Naing N. N. (2002). Sociodemographic characteristics of food handlers and their knowledge, attitude and practice towards food sanitation: a preliminary report. *Southeast Asian Journal of Tropical Medicine and Public Health*.

[24] Food and Agriculture Organization The State of Food Insecurity in the World 2006. http://www.fao.org/docrep/009/a0750e/a0750e00.htm.

[25] Food and Agriculture Organization The State of Food Insecurity in the World 2010. http://www.fao.org/docrep/013/i1683e/i1683e.pdf.

[26] Barro N., Bello A. R., Itsiembou Y. (2007). Street-vended foods improvement: contamination mechanisms and application of food safety objective strategy: critical review. *Pakistan Journal of Nutrition*.

[27] Muinde O. K., Kuria E. (2005). Hygienic and sanitary practices of vendors of street foods in Nairobi, Kenya. *African Journal of Food, Agriculture, Nutrition and Development*.

[28] Mensah P., Amar-Klemesu M., Hammond A., Haruna A. (2001). Bacterial contamination on lettuce, tomatoes, beef and goat meat from metropolitan Accra. *Ghana Medical Journal*.

[29] Sheth M., Gurudasani R., Mudbidri R. (2005). Identification of hazards in street foods of Vadodara, India. *Indian Journal of Nutrition and Dietetics*.

[30] Tambekar D. H., Jaiswal V. J., Dhanorkar D. V., Gulhane P. B., Dudhane M. N. (2008). Identification of microbiological hazards and safety of ready-to-eat food vended in streets of Amravati City, India. *Journal of Applied Bioscience*.

[31] Schelin J., Wallin-Carlquist N., Cohn M. T., Lindqvist R., Barker G. C., Rådström P. (2011). The formation of *Staphylococcus aureus* enterotoxin in food environments and advances in risk assessment. *Virulence*.

[32] Schmidt R. H., Renée M. G., Douglas L., Archer, Keith R. S. General Overview of the Causative Agents of Foodborne Illness. http://edis.ifas.ufl.edu/fs099.

[33] Vaillant V., De Valk H., Baron E. (2005). Foodborne infections in France. *Foodborne Pathogens and Disease*.

[34] Nyenje M. E., Odjadjare C. E., Tanih N. F., Green E., Ndip R. N. (2012). Foodborne pathogens recovered from ready-to-eat foods from roadside cafeterias and retail outlets in Alice, Eastern Cape Province, South Africa: public health implications. *International Journal of Environmental Research and Public Health*.

[35] Donkor E. S., Kayang B. B., Quaye J., Akyeh M. L. (2009). Application of the WHO keys of safer food to improve food handling practices of food vendors in a poor resource community in Ghana. *International Journal of Environmental Research and Public Health*.

[36] Tambekar D. H., Gulhane S. R., Jaisingkar R. S., Wangikar M. S., Banginwar Y. S., Mogarekar M. R. (2008). Household Water management: a systematic study of bacteriological contamination between source and point-of-use. *American-Eurasian Journal of Agricultural and Environmental Science*.

[37] Roberts K. R., Barrett B. B., Howells A. D., Shanklin C. W., Pilling V. K., Brannon L. A. (2008). Food safety training and foodservice employees' knowledge and behavior. *Food Protection Trends*.

[38] Teplitski M., Barak J. D., Schneider K. R. (2009). Human enteric pathogens in produce: un-answered ecological questions with direct implications for food safety. *Current Opinion in Biotechnology*.

